# Targeting the Tumour Vasculature: Exploitation of Low Oxygenation and Sensitivity to NOS Inhibition by Treatment with a Hypoxic Cytotoxin

**DOI:** 10.1371/journal.pone.0076832

**Published:** 2013-10-28

**Authors:** Jennifer H. E. Baker, Alastair H. Kyle, Kirsten L. Bartels, Stephen P. Methot, Erin J. Flanagan, Andrew Balbirnie, Jordan D. Cran, Andrew I. Minchinton

**Affiliations:** Integrative Oncology – Radiation Biology Unit, BC Cancer Research Centre, Vancouver, British Columbia, Canada; Ospedale Pediatrico Bambino Gesu', Italy

## Abstract

Many cancer research efforts focus on exploiting genetic-level features that may be targeted for therapy. Tissue-level features of the tumour microenvironment also represent useful therapeutic targets. Here we investigate the presence of low oxygen tension and sensitivity to NOS inhibition of tumour vasculature as potential tumour-specific features that may be targeted by hypoxic cytotoxins, a class of therapeutics currently under investigation. We have previously demonstrated that tirapazamine (TPZ) mediates central vascular dysfunction in tumours. TPZ is a hypoxic cytotoxin that is also a competitive inhibitor of NOS. Here we further investigated the vascular-targeting activity of TPZ by combining it with NOS inhibitor L-NNA, or with low oxygen content gas breathing. Tumours were analyzed via multiplex immunohistochemical staining that revealed irreversible loss of perfusion and enhanced tumour cell death when TPZ was combined with either low oxygen or a NOS inhibitor. Tumour growth rate was reduced by TPZ + NOS inhibition, and tumours previously resistant to TPZ-mediated vascular dysfunction were sensitized by low oxygen breathing. Additional mapping analysis suggests that tumours with reduced vascular-associated stroma may have greater sensitivity to these effects. These results indicate that poorly oxygenated tumour vessels, also being abnormally organized and with inadequate smooth muscle, may be successfully targeted for significant anti-cancer effects by inhibition of NOS and hypoxia-activated prodrug toxicity. This strategy illustrates a novel use of hypoxia-activated cytotoxic prodrugs as vascular targeting agents, and also represents a novel mechanism for targeting tumour vessels.

## Introduction

Identification of tumour-specific, targetable features for which effective anti-cancer therapeutics can be generated is an important focus in cancer research. The variable tumour microenvironment presents opportunities for chemotherapeutic damage, with targets including hypoxic cells and the abnormal tumour vasculature.

The presence and importance of hypoxia in tumours has been recognized for more than 50 years [1]. The supply of oxygen to tumours is compromised by low microvessel density, abnormal vascular architecture, low blood oxygenation and slow or stagnant blood flow [2]. Tirapazamine (TPZ; SR4233; 3-amino-1,2,4-benzotriazine 1,4-dioxide) is a hypoxic cytotoxin thought to specifically damage poorly oxygenated tumour cells [3]. Cellular reductases, including nitric oxide synthase (NOS), reduce and bioactivate TPZ, and in the absence of oxygen TPZ is further metabolized to oxidizing radicals capable of causing DNA damage [4]. TPZ has greater toxicity to hypoxic cells than to oxygenated cells *in vitro* and enhances cell kill by radiotherapy and cisplatin *in vivo* [5], [6], [7]. However, TPZ has also demonstrated an unexpected ability to effect a dose-dependent, large area of vascular dysfunction in the central regions of tumour xenografts [8], [9], [10]. TPZ-mediated vascular dysfunction occurs in only 65% of treated tumours, is characterized by loss of perfusion that leads to death of the dependent tumour cells, and leaves a hallmark viable rim of undamaged vessels in affected tumours. In previous work using DCE-MRI biomarkers we have shown that tumours with greater pre-treatment vascular function are less responsive to TPZ-mediated vascular damage [8].

The pattern of central vascular damage and persistence of a viable rim caused by TPZ is similar to that seen for vascular disrupting agents (VDAs). Distinct from anti-angiogenic agents, VDAs are a class of compounds in clinical trial that aims to target the aberrant nature of existing tumour blood vessels (for a review regarding VDAs see [11]). VDAs cause a loss of perfusion in existing tumour vessels that is followed by large areas of necrosis, typically with a characteristic surviving viable rim of functioning vessels and viable tissue. VDAs work either by a ligand-based mechanism that targets toxic drugs to antigens expressed specifically on tumour vascular endothelium, or by interfering with the cytoskeleton of tumour endothelial cells to cause acute changes in tumour vascular permeability [12], [13]. Tumour blood vessel features that have been attributed to be at least partially responsible for the specific sensitivity to VDAs include a higher than normal interstitial fluid pressure [14], poor vascular architecture that leads to high permeability and low blood flow [2], [15], as well as the fragility of immature vessels with poor coverage of mural support cells [16], [17].

The pattern of centralized vascular damage similar to that for VDAs, as well as the role of vascular function as a possible predictor for tumour response to TPZ, have prompted us to investigate the role of microenvironment features in TPZ sensitivity. The disappointing results for TPZ in clinical trials [3], [18], [19], makes it relevant to investigate this alternate mechanism for TPZ activity *in vivo*. Here we examine details of the tumour microenvironment mapped using multiplexed immunohistochemistry to identify potential indicators of sensitivity. TPZ has known aerobic toxicity [20], therefore it is useful to investigate and determine whether the anti-vascular effects of TPZ are related to its hypoxic cytotoxicity. To our knowledge, no other studies have evaluated hypoxic vasculature as a possible targetable feature for tumour-specific damage. We also use tumour mapping to evaluate the tumour microenvironment following TPZ in combination with a NOS inhibitor in multiple models. TPZ and other hypoxic cytotoxins are non-competitive inhibitors of NOS [21], [22], the enzyme responsible for generating NO, a critical molecule for endothelial cell signalling. Lastly, we are able to demonstrate inter- and intra- tumour heterogeneity in many elements of the microenvironment, some of which appear to be linked to sensitivity to the VDA activity of the hypoxic cytotoxin TPZ.

## Materials and Methods

### Mice and tumours

Ethics Statement: Experiments described in this manuscript were approved by the Animal Care Committee of the University of British Columbia in accordance with the Canadian Council on Animal Care guidelines. Female NOD/SCID and C3H/HeN mice were bred and maintained in our institutional SPF animal facility. Hypoxia was induced in mice breathing humidified 7 or 10% O_2_/5% CO_2_/balance N_2_ for 30 min prior to drug administration and for 6 h post administration similar to previously used methods [23]. Cells were maintained as monolayers using minimum essential media, MEM/EBSS (HyClone) supplemented with 10% bovine growth serum (HyClone) and passaged every 3 to 4 days. HCT116 (8×10^6^ cells, in NOD/SCID mice), HT29 (5×10^6^ cells, in NOD/SCID) and SCCVII (5×10^5^ cells, in C3H/HeN) were implanted subcutaneously into the sacral region of mice. HCT116 and HT29 cells were purchased from the ATCC; SCCVII, murine squamous carcinoma cells, were obtained in 1983 from Dr. M. Horsman (Stanford University) and held in liquid nitrogen storage. For tumour mapping experiments mice were randomly assigned to experimental groups when average tumour volumes reached 100 to 150 mm^3^ as measured using calipers of three orthogonal diameters (a, b and c) where volume  = π/6 (abc). Average tumour mass at time of excision for each model and group was a minimum of 100 mg and a maximum of 200 mg. For growth assessment tumours were randomly assigned for treatment with 40 mg/kg TPZ, 180 mg/kg L-NNA or a combination of both drugs at 14 days post-implantation when tumours measured 59.0±7 mm^3^; repeat measurements were performed blind.

### Reagents

Tirapazamine was synthesized by Dr. L.A. Huxham [9] and administered at 60 or 40 mg/kg (0.34 or 0.23 mmol/kg) by intraperitoneal (i.p.) injection. L-NNA (Sigma) was administered at 180 mg/kg by i.p. injection. The S-phase marker 5-Bromo-2-deoxyuridine (BrdUrd, Sigma Chemical) was administered at 1000 mg/kg along with 60 mg/kg of the hypoxia marker pimonidazole (provided by Dr. J Raleigh) as an i.p. injection 2 h prior to tissue harvest. Either of the fluorescent dyes DiOC_7_(3) (Molecular Probes), administered as 35 µl of 0.6 mg/ml dissolved in 75% (v/v) dimethyl sulfoxide/25% sterile H_2_O, or 500 kDa FITC-labeled dextran (Sigma), administered as 50 µl of 40 mg/ml solution in NaCl [16], were injected intravenously 5 (DiOC_7_(3)) or 2–20 (FITC-dextran) min prior to euthanasia.

### Immunohistochemistry

Immediately after excision, tumours were frozen on an aluminum block held at −20°C, embedded in cutting medium (OCT, Tissue-TEK) and stored at −20°C until sectioning. The general immunohistochemical procedure used has been previously reported [9]. Briefly, 10 µm tumour cryosections were obtained 2–3 mm from the tumour edge, were air-dried, imaged for native DiOC_7_(3) or FITC-dextran fluorescence and fixed in 50% (v/v) acetone/methanol for 10 min at room temperature. Vasculature was identified using a MAb to PECAM/CD31 (BD PharMingen), bound pimonidazole using Hypoxyprobe^TM^-1 MAb1, eNOS using a mouse MAb (Pharmingen), non-isoform specific NOS using a rabbit PAb to universal NOS (uNOS; NeoMarkers); basement membrane using a rabbit PAb to CIV (NeoMarkers); smooth muscle using a mouse MAb to αSMA (NeoMarkers) and incorporated BrdUrd using a mouse MAb to clone BU33 (Sigma), with light counterstaining using 25% hematoxylin (Sigma).

### Image acquisition and overlay

The imaging system has been previously described [24]. Images of entire tumour cryosections were captured at a resolution of 0.75 µm per pixel. Grayscale CD31 and DiOC_7_(3) images were thresholded and a composite colour image was produced (CD31 alone in red, or overlapped with DiOC_7_(3) in dark blue prioritized over DiOC_7_(3) alone in cyan) using Adobe Photoshop CS (version 8.0). The combined vascular image was then overlaid onto grayscale images of BrdUrd and hematoxylin, where converted grayscale images of pimonidazole (green channel) were then added using the *multiply* mask. Similarly, grayscale images of CD31 were thresholded and prioritized as an overlay (red) on grayscale images of FITC-dextran. CIV and αSMA are shown as black in original grayscale images with a grey hematoxylin counterstained background.

### Image analysis

Using the ImageJ software application and user-supplied algorithms, fluorescent images were inverted and combinations of FITC-dextran, DiOC_7_(3), CD31, pimonidazole, eNOS, uNOS, BrdUrd and hematoxylin images from each tumour section were aligned, cropped to tumour tissue boundaries and staining artifacts removed. Necrosis was cropped away based on hematoxylin stained sections and the remaining viable fraction (VF) of tumours was calculated based on the ratio of the total number of pixels in necrosis-cropped images by the total number of pixels in whole tumour areas. Percent positive staining was obtained using the proportion of pixels at intensities meeting or exceeding a threshold value above background. Average intensity values represent the average pixel intensity for the whole tumour cropped to viable tissue boundaries. For distribution analysis of pimonidazole or FITC-dextran relative to vasculature, each pixel in an image was sorted based on its distance relative to the nearest CD31-positive vessel and the average intensity in 1.5 µm increments from vasculature was determined. For dual positive staining analysis of CD31 in combination with additional markers, thresholds were set to identify staining above background and a minimum 20% overlap was required to classify CD31 objects as dual labeled. The proportion of perfused (PF) and eNOS +ve vessels was obtained by dividing the total number of CD31 objects also positive for DiOC_7_(3) or eNOS respectively by the total number of CD31 objects.

### Vascular Dysfunction Score (VDS)

The VDS score has previously been reported [8] and was used again here with a modification:
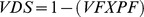
where VF (viable fraction) and PF (perfused fraction) are calculated as described above. A value of 0 indicates 100% viable tissue with perfused vasculature, whereas a value of 1 indicates complete vascular dysfunction, where both the VF and PF are 0. This calculated score is necessary, as loss of functional vasculature may manifest as unperfused vessels and/or as necrotic tissue if the tumour cells have died as a result of reduced blood flow. Necrosis also exists in control tumours, therefore neither measure (VF or PF) may independently reflect the degree of change in perfusion as a result of treatment. The VDS is calculated independently for each tumour and these values are then compared to the VDS_min_ calculated as the control mean plus two standard deviations of the mean (2× SD). This comparison allows for an objective and quantitative detection of unperfused vessels and necrotic tissue significantly greater than that seen in control tumours. Tumours that both scored higher than their control VDS_min_ and showed focused areas of vascular dysfunction in tumour maps were considered positive for vascular dysfunction.

### Endothelial Tube Assay

Plates (24 well, Fisher) were coated in 200 µl of 50% Matrigel (BD Pharmingen) in MCDB-131 media and allowed to set at 37°C for 1–2 h. Human microvascular endothelial cells (HMEC; provided by Dr. Aly Karsan [25], [26]) were seeded (1×10^5^ cells/well) in 1.5 ml MCDB-131 media (Sigma) supplemented with 10% FBS, 10 µg/ml EGF (Sigma) and 10 ng/ml glutamine (Sigma). Cells formed tubular structures by 24 h post-seeding and were subsequently treated at indicated concentrations of TPZ; minimum 5 wells per treatment. Plates were brought to indicated gas concentrations in 10.3 min using a custom built aluminum chamber sealed and equilibrated via 8×1 min cycles of evacuation and pressurization and final pressure was maintained for 1 h at 37°C. Wells were then rinsed and the media replaced with supplemented MCDB-131 and were incubated at 37°C in 5% O_2_/5% CO_2_/90% N_2_. Fluorescent dye cell viability indicator Calcein AM (Invitrogen), 10 µM in media, was added 1 h prior to endpoint and was rinsed with phosphate buffered saline (PBS, HyClone) prior to fixation in 10% formalin (Sigma) overnight.

### Statistics

All statistical analyses were performed using GraphPad Prism software (version 4.0e for Macintosh). Nonparametric Mann-Whitney U tests were used for comparisons between groups; p values *<0.05, **<0.01 and **<0.001 are reported. Where appropriate, charts display values for individual tumours as means for analysis of whole tumour sections; combined means are reported for 4–8 tumours per group ± standard error (s.e.).

## Results

### HT29 colorectal xenografts are resistant to vascular dysfunction effects of TPZ

**Figure 1:**
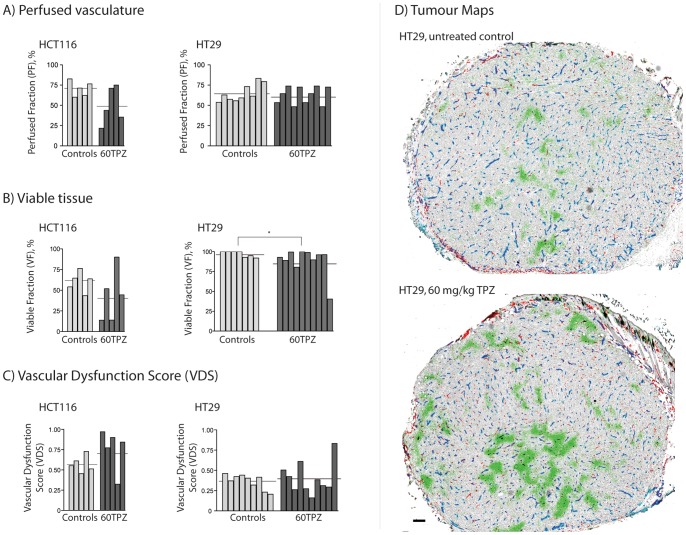
HT29 and HCT116 colorectal xenograft sensitivity to anti-vascular effects of TPZ. HCT116 and HT29 colorectal xenografts were collected 24/kg TPZ. Individual tumour values (bars) and group means (horizontal lines) are show for (A) the perfused fraction (PF), (B) viable fraction (VF) and (C) combined VDS. (D) Representative images of control (top) and 60 mg/kg TPZ-treated (bottom) HT29 colorectal xenografts; staining shows unperfused vasculature (CD31, red), perfused blood vasculature (CD31 overlapped with DiOC_7_(3), blue), perfusion dye (DiOC_7_(3), cyan), hypoxia (pimonidazole, green), S-phase cells (BrdUrd, black) overlaid on hematoxylin background staining (grey). Scale bar 150 µm; (*p<0.05).

HT29 xenografts treated with TPZ show no difference in their proportion of perfused vasculature (PF), however a small but significant decrease in the proportion of viable tissue (VF) is seen (Fig. 1A, B; Table 1); mass for controls and TPZ-treated tumours is 163.5±22.5 mg and 161.5±7.4 mg respectively. Only 1 of 10 HT29 tumours scored a VDS value > VDS_min_ (0.505) (Fig. 1C). HT29 tumour maps confirm no evidence of central vascular damage in response to TPZ, with exception to the single tumour scoring > VDS_min_ which has a central area of necrosis; representative images are shown (Fig. 1D). TPZ treatment in HCT116 xenografts resulted in 3 of 5 tumours exhibiting vascular dysfunction and scoring > VDS_min_ (0.782), although no significant differences were found in PF and VF values between groups (Fig. 1C, Table 1), likely due to the intra-group heterogeneity; mass for controls and TPZ-treated tumours is 186.8±28.0 mg and 162.2±20.9 mg respectively. This frequency of response is consistent with that reported previously for HCT116 tumours, which have been found to consistently be sensitive to TPZ-mediated vascular dysfunction at this rate of sensitivity [8], [9]. HCT-116 tumours are thus described as “sensitive” to these TPZ vascular effects compared to the “resistant” HT29 tumours.

**Table 1:** Quantified measurements of Vascular Dysfunction in HT29 & HCT116 tumours treated with TPZ.

Group	PF% ± SD	VF% ± SD	VDS ± SD	VDS_min_	# tumours > VDS_min_
**HT29**					
Control	65.2±10.8	97.8±3.4	0.364±0.09	0.549	n/a
60TPZ	65.3±13.4	88.4±17.9	0.406±0.20	-	1/10
**HCT116**					
Control	70.6±9.5	60.1±12.4	0.572±0.10	0.782	n/a
60TPZ	49.5±23.0	42.8±31.6	0.762±0.26	-	3/5

Please see Figure 1 for statistical analyses. PF  =  perfused fraction; SD  =  standard deviation; VF  =  viable fraction; VDS  =  vascular dysfunction score; VDS_min_  =  (mean control VDS) + (2× SD); 60TPZ  =  tirapazamine at 60 mg/kg.

### Differences in vascular phenotype and function between HCT116 and HT29 colorectal xenografts

**Figure 2:**
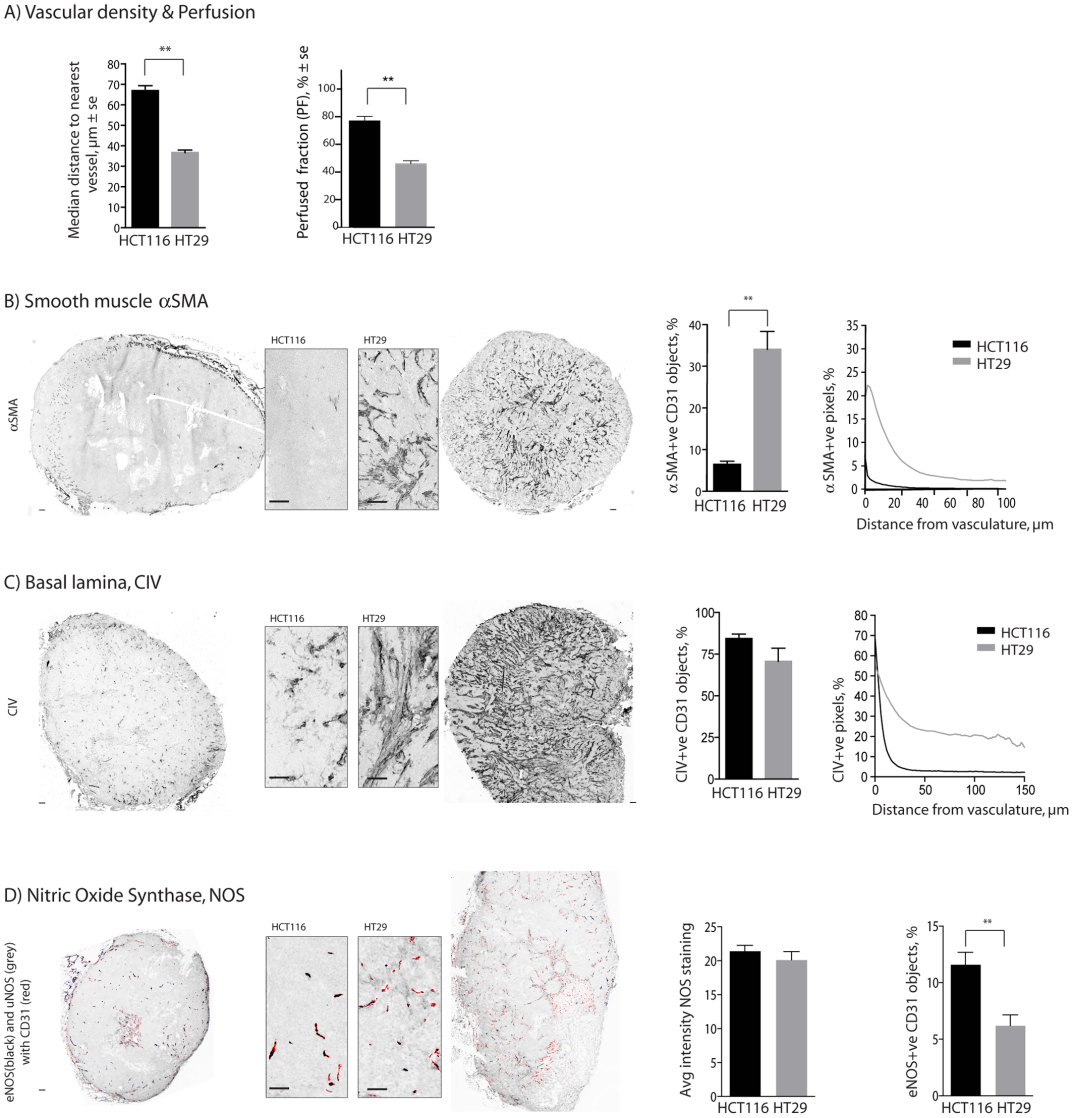
Vascular architecture of HCT116 and HT29 colorectal xenografts. Differences are observed between HCT116 and HT29 colorectal xenografts for vessel density (A, left) and perfused fraction (A, right). The amounts of smooth muscle (SMA, B) and basal lamina (CIV, C) are assessed as the fraction of vessels dual labeled for the marker as well as the amount of staining as a function of distance from nearest vessels. Nitric Oxide Synthase (NOS) staining (D) is shown as the average amount of universal NOS (uNOS, grey) in all viable tissue or as the fraction of vessels (CD31 alone, red; overlapped with eNOS, black) dual labeled for endothelial NOS (eNOS) (overlapped with CD31, black). Whole tumour images and higher resolution inserts illustrate the micro-regional heterogeneity in HCT116 & HT29 tumours; scale bars 150 µm; (**p<0.01).

HCT116 and HT29 are both colorectal xenografts, which grow at similar rates in identical mouse models with genetically identical endothelium, suggesting that features of the tumour microenvironment are responsible for the differential sensitivity of HCT116 and HT29 tumours. The distance between vessels is higher in HCT116s, meaning a *lower* vascular density (Fig. 2A, left), while the proportion of perfused vessels is significantly lower in HT29 tumours relative to HCT116 (Fig. 2A, right). The proportion of vessels with overlapping smooth muscle cells is significantly greater in HT29 tumours, where thicker layers of aSMA are seen proximal to vasculature (Fig. 2B). The proportion of vessels with collagen type IV basal lamina (CIV) overlap was similar in both HCT116 and HT29 colorectal xenograft models, however, a greater amount of is found throughout the HT29 tumours (Fig. 2C). Whole tumour maps illustrate these quantitative analyses, where it is clear that a significantly greater proportion of HT29 xenografts are comprised of stromal tissue, much of which is proximal to vasculature.

Expression of all isoforms of NOS, reflected by staining intensity data from whole tumour sections stained for uNOS, is similar in HCT116 and HT29 (Fig. 2D). Specific expression of eNOS staining is expressed as the proportion of CD31 +ve vessels that are at minimum 20% dual labeled for eNOS staining. HT29 tumours have significantly fewer eNOS+ve CD31 vessels relative to HCT116 tumours. However, as HT29 tumours have a greater overall vascular density, there is no significant difference in the overall eNOS+ve CD31 vessel density between tumours; HT29 tumours have a greater number of eNOS-ve vessels. Average tumour mass for HCT116 and HT29 tumours for Fig. 2A-2D is 152.6±11.5 mg and 178.0±27.9 mg respectively.

**Figure 3:**
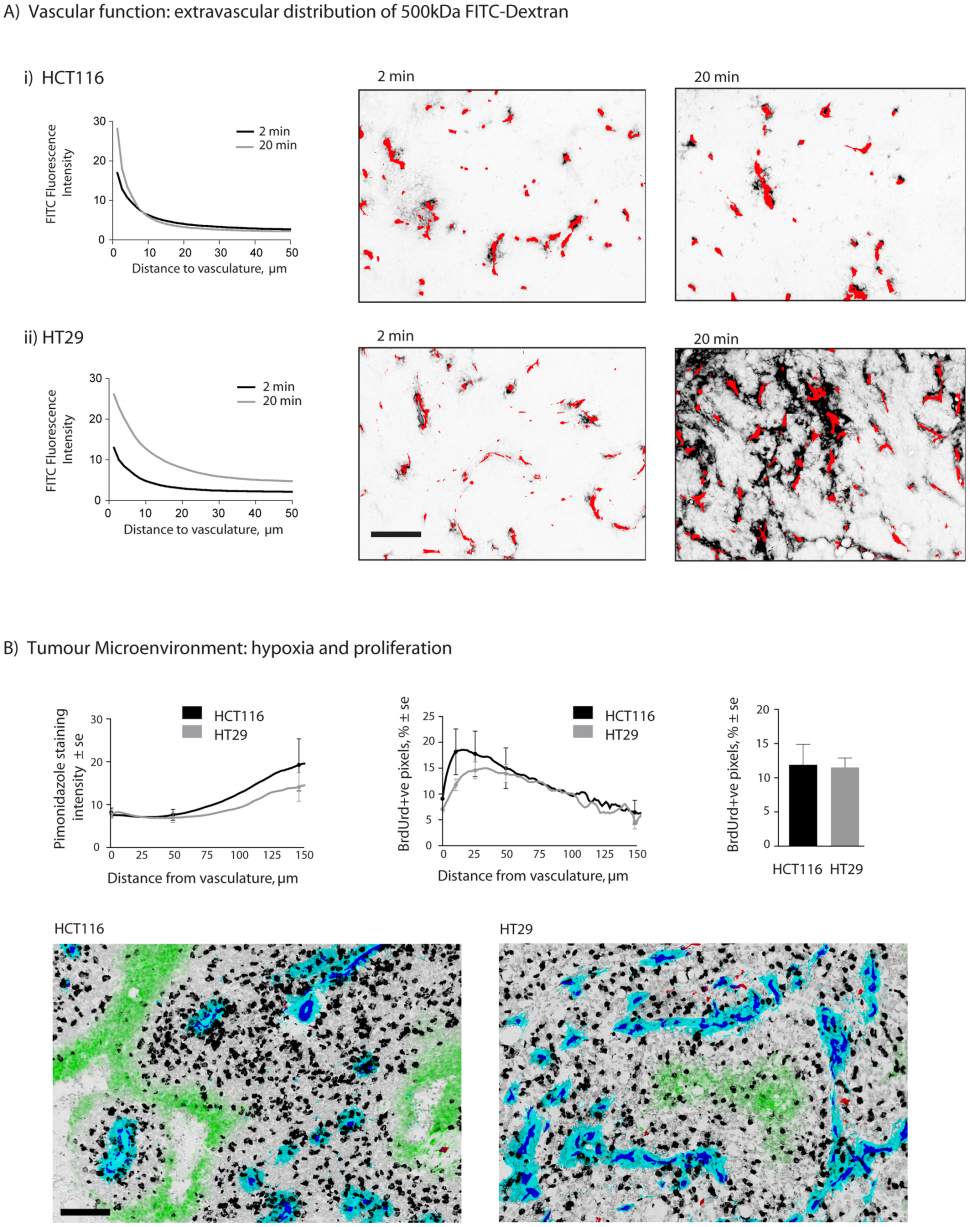
Vascular function and tumour microenvironment in HCT116 and HT29 colorectal xenografts. HCT116 & HT29 tumours were harvested at 2 or 20 min after intravenous injection of 500 kDa FITC-dextran (A). Fluorescence intensity of FITC-labeled dextran is displayed as a function of distance from nearest vasculature in HCT116 (i) and HT29 (ii) xenografts. Images show FITC fluorescence (black) and CD31 stained vasculature (red). Quantitative analysis of the tumour microenvironment (B) shows incorporated BrdUrd staining as a marker for S-phase cells as whole tumour means (B, right) or as a function of distance from nearest vessel (B, middle). Tumour hypoxia is reflected by pimonidazole labeling as a function of distance from vasculature (B, left). Magnified tumour map images reflect representative staining patterns of HCT116 and HT29; staining shows unperfused vasculature (CD31, red), perfused blood vessels (CD31 overlapped with DiOC_7_(3), blue), perfusion dye (DiOC_7_(3), cyan), hypoxia (pimonidazole, green) and s-phase (BrdUrd, black) overlaid on hematoxylin background staining (grey). Scale bars 150 µm.

Quantitative analysis of the presence of 500 kDa FITC-dextran as a function of distance from nearest blood vessels is interpreted as a measure of vessel leakiness; greater amounts of the macromolecule found in the perivascular area indicates that more of it was able to leak from vessels. These data are distinct from measurements of perfusion status using the small molecule carbocyanine (Fig. 2A), which is only in the blood stream for 5 minutes, such that it labels those vessels that were patent at the time of tissue harvest. In HCT116 the intensity of FITC at distances farther from vasculature is similar when examined at 2 and 20 min post i.v. administration (Fig. 3Ai). However, in the HT29 model greater fluorescence is seen at greater distances from vasculature with time (Fig. 3Aii), suggesting greater extravasation and extra-vascular distribution of the high MW marker in HT29 tumours. Average tumour mass for HCT116 and HT29 tumours for Fig. 3A-3B is 166.2±17.8 mg and 181.6±22.4 mg respectively.

### Microenvironmental differences between HCT116 and HT29 colorectal xenografts

Proliferating cells and hypoxia were also mapped in untreated HCT116 and HT29 colorectal xenografts. The overall proportion of S-phase cells is reflected by the % pixels positive for incorporated BrdUrd, where no difference is seen between models (Fig. 3B, right). However, HT29 xenografts show a trend of fewer proliferating cells proximal to vasculature compared to HCT116 (Fig. 3B, middle). The amount of bound pimonidazole is somewhat lower in HT29 tumours, particularly at distances further from vasculature (>75 µm) (Fig. 3B, left), but both are predominantly characterized by diffusion-limited hypoxia, as shown by the presence of pimonidazole-labeled hypoxia predominantly located at distances far from vasculature. Very little evidence of intermittent perfusion related hypoxia is observed, as shown by the absence of the hypoxia marker label overlapping blood vessels; for a more detailed description of different types of hypoxia please see [27]. Sample images of the tumour microenvironment are shown in Fig. 3B.

### NOS inhibition enhances TPZ-mediated vascular dysfunction and growth inhibition effects of TPZ in HCT116 xenografts

**Figure 4:**
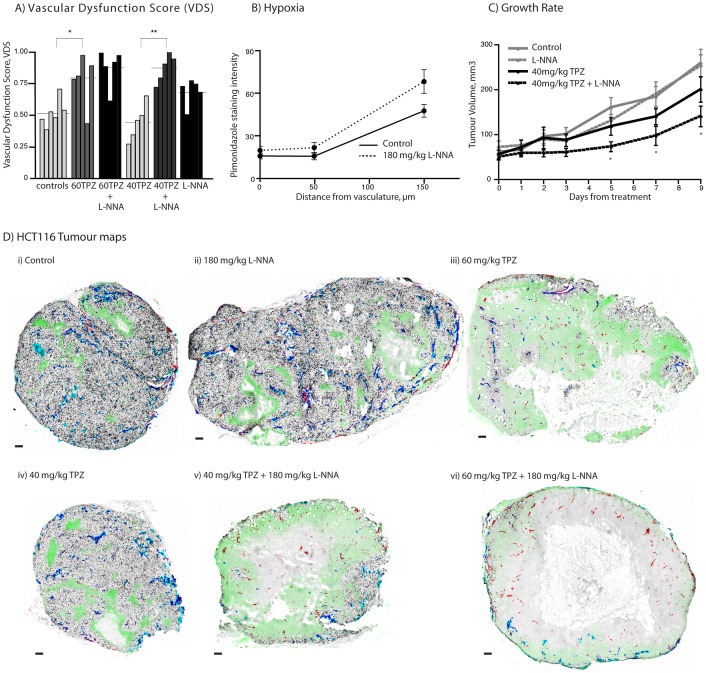
NOS inhibition enhances anti-vascular effects of TPZ in HCT116 xenografts. HCT116 colorectal xenografts were treated with TPZ at 40/kg or 60 mg/kg alone or in combination with 180 mg/kg NOS inhibitor L-NNA. (A) VDS is reported for individual tumours (bars); horizontal lines represent group means. (B) graph shows staining intensity of pimonidazole as a function of distance from vasculature, showing micro regional differences in hypoxia for control and L-NNA treated tumours, with no significant differences. (C) Tumour growth rate data for control and single or combination treated groups; each point represents mean ± SE. (D) Tumour maps show staining for unperfused vasculature (CD31, red), perfused vasculature (DiOC7 (3)+ve CD31, blue), S-phase cells (BrdUrd, black) and hypoxia (pimonidazole, green). Tumours representative of central vascular dysfunction effects observed within indicated treatment groups are shown for each group; (*p<0.05) (**p<0.01); scale bars 150 µm.

HCT116 xenografts treated with high (60 mg/kg) and low (40 mg/kg) dose TPZ and L-NNA alone or in combination were assessed; VDS scores as well as perfusion and necrosis values are shown in Table 2. HCT116 tumours treated with a combination of L-NNA and 40 mg/kg TPZ resulted in an increased anti-vascular response at 24 h relative to TPZ alone (Fig. 4A). Average tumour mass for control, 60TPZ, 60TPZ + L-NNA, 40TPZ, 40TPZ + L-NNA and L-NNA alone were: 125.2±26.0, 132.6±23.9, 112.2±13.5, 122.4±8.3, 118.0±16.6, 114.4±11.0 respectively. Tumour map images show representative vascular dysfunction from each treatment group (Fig. 4D) where tumours from the 60TPZ (iii), 40TPZ+L-NNA (v) and 60TPZ+L-NNA (vi) are all shown to have loss of perfusion and increased necrosis in the central regions. Some tumours treated with L-NNA alone showed a reduced perfused fraction (PF) relative to untreated controls, consistent with other reports of loss of perfusion in tumours treated with a NOS inhibitor [28], [29], [30], [31]. However, this perfusion change was distributed throughout the tumour and did not result in a centralized loss of blood flow and secondary tumour cell death (representative tumour map shown Fig. 4D (ii)). Hypoxia, as measured by staining for bound pimonidazole, suggests greater hypoxia at distances far from vasculature for L-NNA treated tumours, consistent with a small loss of perfusion (Fig. 4B). Note that in regions of centralized loss of perfusion a lack of pimonidazole staining is an experimental artifact, deriving from the exogenously administered label requiring functioning vessels to be delivered to the hypoxic regions for binding. In areas with intermittent perfusion pimonidazole is able to diffuse far enough to label chronically hypoxic cells due to the presence of other, neighbouring perfused vessels. However, in large regions without blood flow pimonidazole characteristically labels in a ring at the margins of viable tissue, such as that seen in Fig. 4D (v, vi); this pattern of labeling is a hallmark of central vascular dysfunction seen with tumour maps.

**Table 2:** Quantified measurements of Vascular Dysfunction in tumours treated with TPZ and NOS inhibitor L-NNA.

Group	PF% ± SD	VF% ± SD	VDS ± SD	VDS_min_	# tumours > VDS_min_
**HCT116**					
Control	64.7±8.4	74.0±12.7	0.519±0.11	0.734	n/a
60TPZ	39.7±18.0	46.5±26.8	0.779±0.21	-	4/5
60TPZ + 180L-NNA	30.1±24.3	32.0±22.9	0.878±0.15	-	4/5
40TPZ	63.4±7.4	86.6±16.9	0.446±0.15	-	0/5
40TPZ + 180L-NNA	27.5±18.3	35.5 19.4	0.873±0.11	-	4/5
180L-NNA	48.4±7.9	63.9±14.6	0.688±0.11	-	2/5
**SCCVII**					
Control	27.1±7.5	99.3±1.5	0.731±0.07	0.878	n/a
60TPZ	20.0±12.1	73.3±39.2	0.817±0.13	-	2/5
60TPZ + 180L-NNA	0.62±0.61	1.2±1.2	0.999±0.00	-	5/5
40TPZ	27.3±7.1	95.7±3.3	0.740±0.06	-	0/4
40TPZ + 180L-NNA	16.6±18.9	26.1±28.9	0.916±0.12	-	3/4
180L-NNA	17.1±9.1	77.7±35.1	0.868±0.11	-	3/4
**HT29**					
Control	57.5±4.0	100±0	0.425±0.40	0.505	n/a
60TPZ	62.6±11.3	92.4±8.1	0.415±0.15	-	1/5
60TPZ + 180L-NNA	45.5±11.5	98.8±2.8	0.551±0.11	-	4/5
180L-NNA	54.8±10.5	98.3±3.2	0.459±0.11	-	2/6

**Figure 5:**
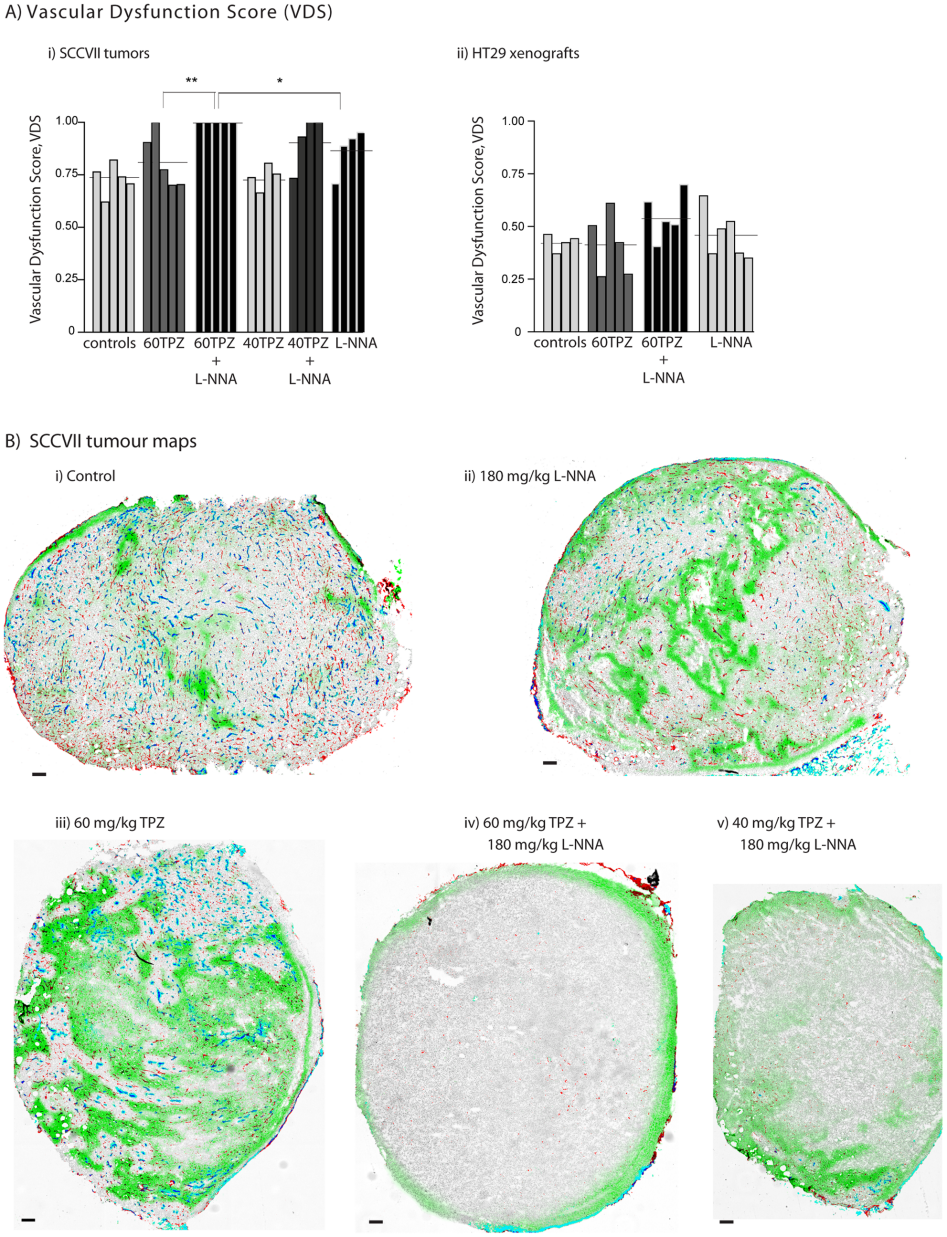
NOS inhibition enhances the anti-vascular effect of TPZ in SCCVII tumours but not HT29 xenografts. HT29 colorectal xenografts and SCCVII murine tumours were treated with 40/kg or 60 mg/kg alone or in combination with 180 mg/kg NOS inhibitor L-NNA. (A) VDS are reported for individual (i) murine SCCVII tumours and (ii) HT29 colorectal xenografts from control and treatment groups (bars); horizontal lines represent group means. (B) Maps of SCCVII tumours show staining for unperfused vasculature (CD31, red), perfused vasculature (DiOC7 (3) +ve CD31, blue), tissue background (hematoxylin, grey) and hypoxia (pimonidazole, green). Tumours representative of central vascular dysfunction effects observed within indicated treatment groups are shown for each group. Scale bars 150 µm; (*p<0.05) (**p<0.01).

Please see Figures 4 and 5 for statistical analyses. PF  =  perfused fraction; SD  =  standard deviation; VF  =  viable fraction; VDS  =  vascular dysfunction score; VDS_min_  =  (mean control VDS) + (2× SD); 60TPZ  =  tirapazamine at 60 mg/kg; 40TPZ  =  tirapazamine at 40 mg/kg; 180L-NNA  = l-nitro-l-arginine at 180 mg/kg.

L-NNA in combination with 40 mg/kg TPZ also results in reduced tumour growth rates relative to that seen for either treatment alone (Fig. 4C). Repeat volume measurements show separation of the combined treatment from all groups, with significant differences relative to untreated controls on days 5, 7 and 9. Mean weight loss was greater in the combination treatment (9.9±2.3%) relative to 40 mg/kg TPZ alone (6.7±1.7%) but this difference was not statistically significant. Higher doses of TPZ or repeat treatments for longer duration were not possible for growth delay studies due to excessive toxicity.

### Differential sensitivity of tumours to vascular dysfunction effects of TPZ alone or in combination with NOS inhibition

SCCVII murine syngeneic tumours have a high vascular density with variable perfusion that results in intermittent hypoxia characterized by the presence of perfused vessels overlapped with hypoxia label pimonidazole. Treatment of SCCVII murine tumours with either L-NNA or TPZ causes a drop in both the PV and VF and produces some focused areas of vascular dysfunction; quantified data for all SCCVII treatment groups are shown in Table 2 and Fig. 5A (i)), tumour maps with representative vascular dysfunction for each group are shown in Fig. 5B. Combining 180 mg/kg L-NNA with 60 mg/kg TPZ dramatically increases the vascular dysfunction response in SCCVII tumours, with the mean VDS score essentially at the maximum of 1. Enhancement of TPZ-mediated vascular response by L-NNA also occurs at lower doses, where 40 mg/kg TPZ alone combined with 180 mg/kg L-NNA results in 3 of 4 tumours showing large areas of vascular dysfunction; representative tumour map shown, Fig. 5B (v). Average tumour mass for control, 60TPZ, 60TPZ + L-NNA, 40TPZ, 40TPZ + L-NNA and L-NNA alone were: 134.2±8.2, 103.8±18.5, 117.2±17.4, 139.8±19.7, 150.8±30.2, 132.2±33.1 respectively.

HT29 colorectal xenografts grow at similar rates relative to HCT116 colorectal xenografts, and are grown in identical mice, but have a higher vascular density. No TPZ-mediated vascular shut down has been observed in HT29 tumours at the 60 mg/kg dose, and combining this treatment with NOS inhibitor L-NNA did not induce a vascular dysfunction response (Fig. 5A (ii)). Quantitative analysis results in 1 of 5 TPZ-treated, 2 of 6 L-NNA treated, and 4 of 5 combination treated tumours scoring a VDS >VDS_min_ (Table 2). However, no large areas of unperfused or necrotic tissues were observed in these tumours, and they are therefore classed as non-responders. Treatment was apparently able to effect loss of vascular function in vessels within the tumours, but these effects were not substantial enough, or were not sufficiently proximal to each other to cause secondary tumour cell death similar to that seen for vascular disrupting agents or for TPZ in other tumour models. This data illustrates the difficulty in assessing tumour vascular dysfunction; it must be assessed *in vivo* and include appreciation of the PF, VF and location of these particular vessels; quantitative and qualitative tumour mapping are optimal for detecting and characterizing vascular damage response in these tumours.

### Lower O_2_ breathing enhances the anti-vascular effects of TPZ in HCT116 and HT29 colorectal xenografts

**Figure 6:**
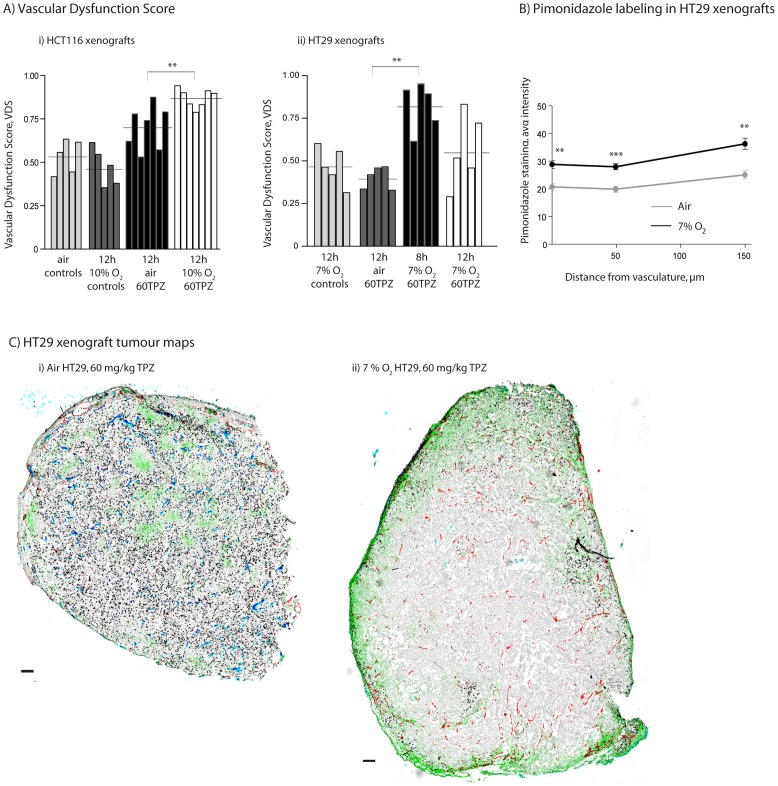
Hypoxia enhances anti-vascular effects of TPZ in HCT116 and HT29 xenografts. HCT116 and HT29 colorectal xenografts were treated with 60/kg TPZ in room air or low oxygen conditions. (A) VDS is reported for individual (i) HCT116 and (ii) HT29 colorectal xenografts (bars) from control and treatment groups in indicated oxygen conditions; horizontal lines represent group means. (B) Staining data shows an increase in hypoxia, via pimonidazole labeling, in low oxygen conditions. (C) Tumour maps of HT29 colorectal xenografts show staining for unperfused vasculature (CD31, red), perfused vasculature (DiOC7 (3) +ve CD31, blue), S-phase cells (BrdUrd, black) and hypoxia (pimonidazole, green). Tumours representative of central vascular dysfunction effects observed within indicated treatment groups are shown for each group; scale bars 150 µm; (*p<0.05) (**p<0.01) (***p<0.001).

HCT116 and HT29 colorectal xenograft bearing mice were exposed to either 10% O_2_/5% CO_2_/balance N_2_ or 7% O_2_/5% CO_2_/balance N_2_ respectively, for 30 min prior and for 6 h post TPZ-administration. Average HCT116 tumour mass for air controls, 12 h 10% O_2_, 12 h air 60TPZ, and 12 h 10% O_2_ TPZ tumours were: 135.4±21.9, 152.6±11.5, 132.5±25.0, 103.5±9.0, 117.2±12.5 respectively. Average HT29 tumour mass for air controls, 12 h 10% O_2_, 12 h air 60TPZ, and 12 h 10% O_2_ TPZ tumours were: 168.4±27.1, 178.0±27.9, 161.2±20.3, 165.8±12.7, 146.8±22.4 respectively. TPZ-treated HCT116 tumours from low pO_2_ mice had a greater magnitude and frequency of vascular dysfunction relative to tumours from mice breathing room air (Fig. 6A, Table 3). As seen in earlier analysis (Fig. 1), HT29 tumours treated with 60 mg/kg TPZ from mice in room air did not sustain vascular dysfunction. However, tumours harvested from mice in reduced oxygen conditions do show central vascular dysfunction with 4 of 5 tumours > VDS_min_ at 8 h and 2 of 5> VDS_min_ at 12 h; qualitative analysis showed centralized, focused areas of loss of perfusion. Lowered O_2_ breathing resulted in increased tumour hypoxia at all distances from vasculature in both tumour models (Fig. 6B); tumours from low oxygen breathing animals had increased amounts of bound pimonidazole at 0 and all distances from vasculature (**p<0.01; ***p<0.001). This effect of hypoxia sensitizing HT29 tumours to the anti-vascular effects of TPZ is demonstrated in representative tumour maps (Fig. 6C).

**Table 3:** Quantified measurements of Vascular Dysfunction in tumours treated with TPZ in reduced Oxygen conditions.

Group	PF% ± SD	VF% ± SD	VDS ± SD	VDS_min_	# tumours > VDS_min_
**HCT116**					
Air Control	67.4±6.6	68.8±10.2	0.534±0.10	0.731	n/a
Air 60TPZ	53.3±9.3	55.0±16.3	0.701±0.13	-	4/7
10% O_2_	66.0±10.9	79.3±8.1	0.475±0.11	-	0/5
10% O_2_ 60TPZ	33.0±12.3	38.9±5.5	0.872±0.54	-	7/7
**HT29**					
Air Control	46.1±5.1	91.5±8.4	0.581±0.03	0.632	n/a
Air 60TPZ, 12h	60.3±6.7	99.1±1.1	0.402±0.07	-	0/5
7% O_2_ Control	55.4±10.3	95.2±4.1	0.471±0.11	-	0/5
7% O_2_ 60TPZ, 8h	28.2±17.8	60.6±19.0	0.823±0.14	-	4/5
7% O_2_ 60TPZ, 12h	49.3±17.8	85.9±19.8	0.564±0.21	-	2/5

Please see Figure 6 for statistical analyses. PF  =  perfused fraction; SD  =  standard deviation; VF  =  viable fraction; VDS  =  vascular dysfunction score; VDS_min_  =  (mean control VDS) + (2× SD); 60TPZ  =  tirapazamine at 60 mg/kg.

### Tirapazamine mediates damage to endothelial tube structures *in vitro* in a time, concentration and oxygen-dependent manner

**Figure 7:**
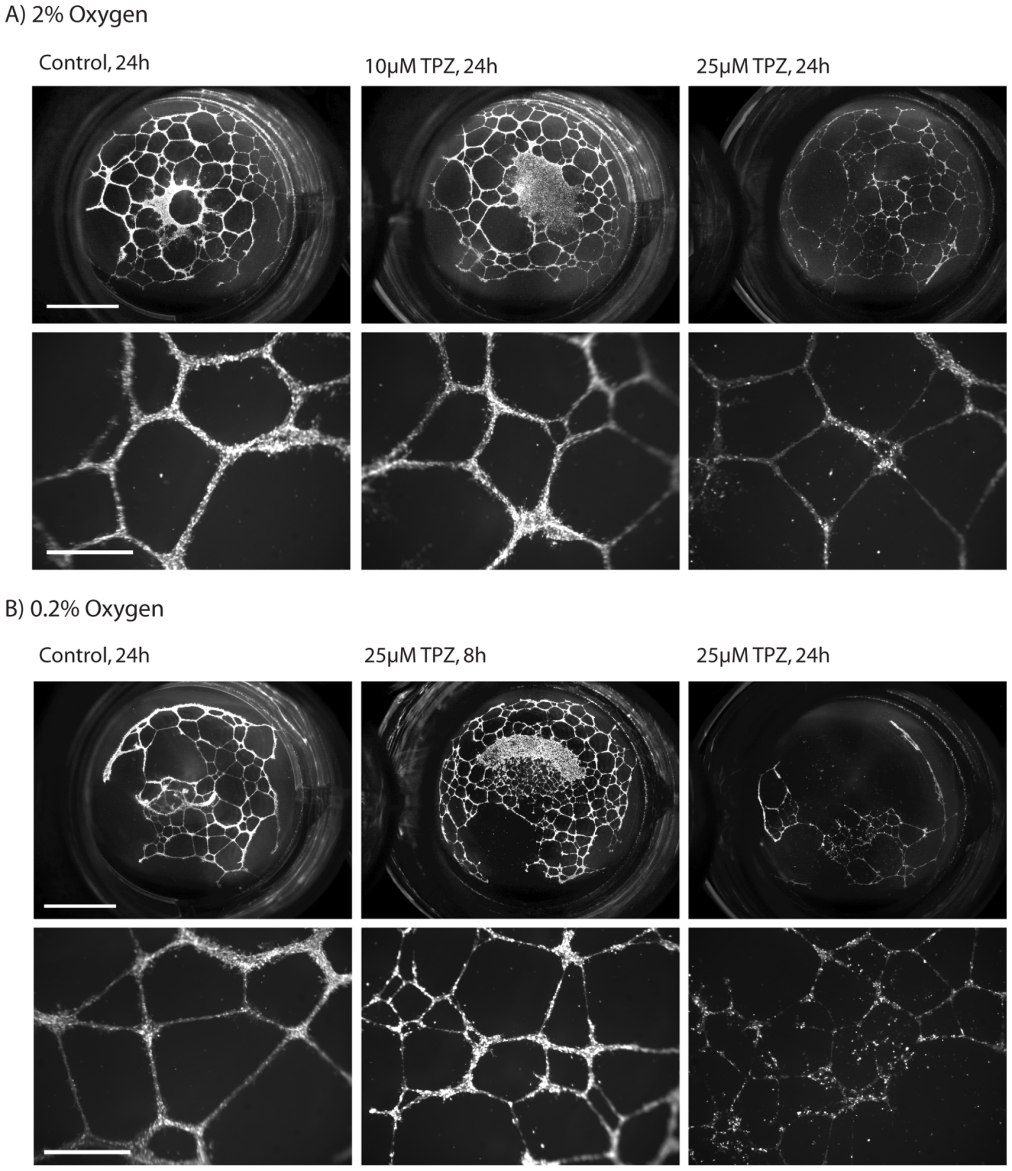
TPZ mediates damage to endothelial tube structures in a concentration, time and oxygen dependent manner. HMECs seeded onto Matrigel-coated plates formed tubular structures by 24 h and were then treated with TPZ in (A) 2% or (B) 0.2% oxygen conditions for indicated times. Whole wells (top; scale bars 500 µm) and magnified representative areas (bottom; scale bars 150 µm) are shown.

HMECs grown as tubular structures on Matrigel-coated 24 well plates were subsequently exposed to TPZ under 2% or 0.2% O_2_/5% CO_2_/balance N_2_ for 1 h, were rinsed, and then 8 or 24 h later were imaged using fluorescent Calcein AM cell viability indicator. Tubular structure networks formed prior to treatment with TPZ are comprised of multiple cells in linear structures. Exposure of the tubes to 10 µM TPZ at 2% O_2_ for 1 h had minimal effect on the network or cellular makeup of the structures, whereas in 25 µM the cell density is reduced, resulting in deteriorated networks (representative images, Fig. 7A). At lower 0.2% O_2_, tubes exposed to 25 µM TPZ were even further deteriorated at 24 h post-treatment, however wells imaged at 8 h post-treatment show a less severe difference in cell density and network structures relative to 24 h (Fig. 7B). These results demonstrate that a 1 h exposure to TPZ at a clinically relevant concentration, 25 µM, in 2% O_2_ can cause vascular damage that does not manifest for several hours post-treatment.

## Discussion

It is counter-intuitive that a hypoxic cytotoxic prodrug would mediate its principle damage to the most highly oxygenated cells in the solid tumour – the tumour vascular endothelium. However, we have previously shown through tumour mapping and MRI studies that TPZ can cause catastrophic damage to the central blood vessels of tumour xenografts, consequently also killing the dependent tumour cells [8], [9], [10]. Here we have extended these studies to demonstrate some microenvironmental features that differ in sensitive vs. resistant tumours, and that hypoxia impacts the sensitivity of tumour vasculature to damage by TPZ. In addition, combining TPZ with a NOS inhibitor results in enhanced damage and anti-cancer activity, representing a new therapeutic strategy. These detailed and comprehensive analyses of tumour features using quantitative and illustrative tumour maps reveal the complexity and heterogeneity of the microenvironment and the difficulties in assessing their unique contributions to observed effects.

Several studies have shown that tumour blood vessels exist at lower than normal oxygen tensions, particularly towards the centre of solid tumours, despite the presence of blood flow [32], [33], [34]. While it is intuitive that greater hypoxia would result in greater damage by a hypoxic cytotoxin, we are looking specifically at damage to the ***tumour vascular endothelium*** under hypoxic conditions, and this has not previously been investigated. This is particularly relevant due to the known effects of TPZ in oxygenated and intermediate oxygen conditions [20]. We have demonstrated that TPZ-induced vascular damage in solid tumours was enhanced by making blood & vasculature more hypoxic through low oxygen breathing (Fig. 6), suggesting that the ***hypoxic*** nature of the vessels may be at least partially responsible for VDA activity of TPZ.

In addition to the importance of oxygen, we have demonstrated greater anti-cancer activity as well as enhanced vascular damaging effects of TPZ combined with a NOS inhibitor, including when TPZ is administered at lower than its maximum dose (Fig. 4). TPZ is enzymatically reduced by NOS, which results in both activation of TPZ and a reduction in produced NO due to competitive inhbition [21]. A reduction in NO levels can have acute effects on tumour vasculature, including decreasing tumour blood flow and contracted vessel diameters [28], [29], [30], [31]. For a comprehensive review of the role of NO in tumour biology, see [35]. Inhibition of NOS has been observed to increase tumour hypoxia and has therefore been suggested as a suitable complementary treatment for hypoxic cytotoxins [36]. Given the observation that TPZ is a competitive inhibitor of NOS, Garner *et al.* (1999) have proposed that TPZ may potentiate itself via this mechanism [21].

The explicit mechanism by which co-administration of a NOS inhibitor enhances the anti-vascular effects of TPZ is beyond the scope of this work. There are several possibilities that all may have contributions to the effect. TPZ produces oxidizing species under both aerobic and anaerobic conditions, as cycling of the prodrug in the presence of oxygen results in O_2_•^−^ as a by-product. In the absence of oxygen, the TPZ radical undergoes further metabolism. The radical intermediate responsible for mediating hypoxic cytotoxicity is thought to be an oxidizing hydroxyl (OH•) or benzotriazinyl radical (BTZ•) [4], [37]. NO interactions with reactive oxygen species (ROS) are complex and often contradictory, having been implicated for both damage and cytoprotection depending on the physiological situation [38], [39], [40]. NO can act as an anti-oxidant, neutralizing reactive oxygen species (ROS). Inhibition of the enzyme NOS either by TPZ alone or by co-administered L-NNA likely produces conditions of reduced NO availability. Therefore, a decrease in locally available anti-oxidant NO could prolong the presence of ROS to result in vascular damage. Alternatively, inhibition of NOS by L-NNA or TPZ may result in destabilized and therefore sensitized vasculature due to modulation of NO levels. Lastly, inhibition of NOS may reduce blood flow, causing decreased blood oxygenation or increased tumour hypoxia to result in enhancement of TPZ hypoxic cytotoxic damage to endothelium. This effect is seen in Figs. 4 and 5 where administration of the NOS inhibitor alone resulted in a drop in the perfused fraction and an increase in tumour hypoxia. Further work is required to determine which of these effects, or what combination of these effects, is relevant to the mechanism for TPZ mediated vascular dysfunction. In particular it is not clear whether combining a NOS inhibitor with TPZ alters the ability of NOS to reduce TPZ; paradoxically, adding a NOS inhibitor could result in less activation of TPZ due to competition at the NOS active site. It is plausible that TPZ mediates vascular damage through a different mechanism when administered alone vs when combined with a NOS inhibitor.

Assessment of an anti-vascular effect is challenging, as shown by the inability of even the VDS, which accounts for both perfusion & necrosis data, to quantitatively identify those tumours with sustained anti-vascular effects. The gold standard efficacy experiment for hypoxic cytotoxins is to combine them with radiation, which preferentially kills oxygenated cells, to produce greater cell kill for the combination treatment. However, this approach is not able to discriminate between cells that die due to their hypoxic status far from vasculature and consequent toxicity of TPZ, and those that may have died due to loss of blood supply. Hypoxic cytotoxicity would kill cells promptly and therefore experiments typically harvest tumours for colony formation assay plating at <12 h. An anti-vascular effect may not be measurable unless excision occurs at >24 h, as any earlier excision would “rescue” the cells that would otherwise have died due to nutrient deprivation. There are some unexplained data that suggest this effect has occurred in other laboratories. Clonogenic survival studies of hypoxic cytotoxin RB6145 in combination with NOS inhibitor L-NNA found several magnitudes greater cell kill than expected for a hypoxic cytotoxic effect when tumours were excised at 24 h [36], [41]. We propose that a large, irreversible vascular damaging effect by RB6145 combined with NOS inhibition may explain these results. Future studies of hypoxia-targeted agents should investigate for *in vivo* vascular damage. Indeed, we suggest that it may be desirable to screen for and identify those agents most capable of causing vascular damage. The fragile, hypoxic tumour vasculature may represent a specific and targetable feature of the tumour microenvironment.

Despite some dramatic increases in vascular damage and even eradication of the viable rim in HCT116 and SCCVII tumours, NOS inhibition combined with TPZ still did not produce vascular damage in HT29 tumours (Fig. 5). HCT116 and HT29 cell lines are both colorectal carcinoma models and both were grown as xenografts in genetically identical mice. The endothelial cells that comprise the vasculature in both these tumours are of the same origin, suggesting that it is the tumour microenvironment that confers differential sensitivity to vascular-specific damage. Both tumour models have been shown to respond to TPZ-mediated cytotoxicity, though these studies did not evaluate an anti-vascular effect [42], [43]. There are likely many genetic differences between the two cancer models that could account for variations in anti-cancer growth inhibition as a result of TPZ. The principle factors mediating sensitivity to the hypoxic cytotoxic effects of TPZ are regarded to be the oxygenation status and the presence of reducing enzymes [44], [45], [46]. We have mapped the presence of hypoxia using pimonidazole (Fig. 4, 6), as well as the amount and location of NOS enzymes in both HCT116 and HT29 tumours (Fig. 2). Tumour mapping of vascular architecture characteristics demonstrated considerable differences in the amounts of vascular-associated stromal tissue, with the more resistant HT29 xenograft possessing greater amounts of both smooth muscle and basal lamina (Fig. 2). The micro regional location of high molecular weight FITC-dextran suggests that the vasculature of HT29 tumours may be more permeable than that of HCT116 tumours (Fig. 3A), which is consistent with DCE-MRI data where increased vascular function predicted for decreased sensitivity to TPZ [8]. Studies with combretastatins have suggested similar findings, where tumours with greater smooth muscle coverage were less vulnerable to VDA damage [16]. Interestingly, in our studies it was the tumour model with greater smooth muscle that demonstrated greater vascular leakiness. Our findings are not unique however, as other groups have also found a correlation between greater smooth muscle and greater extravascular distribution of secondary agents, including chemotherapies [47], [48]. It is plausible that any correlations between vessels, smooth muscle, maturity and leakiness are tumour and site-specific, such that both scenarios are possible in different circumstances.

Finally, we demonstrate effects of TPZ on human microvascular endothelial cells (Fig. 7). The data show that damage is a consequence of dose and oxygenation, and that degradation of tubular structure takes some time to manifest, in contrast to other VDAs where increases in vascular permeability are shown within 30 min [49]. Importantly, damage to the endothelium occurred at intermediate O_2_ (2%), and at a clinically relevant concentration (25 µM) [50].

Although TPZ has failed to show a significant clinical benefit, the concept of hypoxia-activated cytotoxic agents represents an active area of research, including other agents that are also inhibitors of NOS (AQ4N, TPZ analogues) [21], [22]. Clinical trials investigating the efficacy of TPZ have struggled due to toxicity and difficulties in screening patients for hypoxia prior to treatment, but may actually benefit from alternative patient selection approaches [18], [19]. In addition to hypoxia, tumour vasculature and blood oxygenation may represent useful prognostic indicators for improved anti-cancer activity, and further investigation into this potentially overlooked mechanism of activity for hypoxic cytotoxins is warranted. Poorly oxygenated blood vessels may represent a uniquely targetable feature of the tumour microenvironment.
